# Decision Making When Cancer Becomes Chronic: Needs Assessment for a Web-Based Medullary Thyroid Carcinoma Patient Decision Aid

**DOI:** 10.2196/27484

**Published:** 2021-07-16

**Authors:** Danielle Shojaie, Aubri S Hoffman, Ruth Amaku, Maria E Cabanillas, Julie Ann Sosa, Steven G Waguespack, Mark E Zafereo, Mimi I Hu, Elizabeth E Grubbs

**Affiliations:** 1 Department of Surgical Oncology The University of Texas MD Anderson Cancer Center Houston, TX United States; 2 Department of Gynecological Oncology and Reproductive Medicine The University of Texas MD Anderson Cancer Center Houston, TX United States; 3 Department of Endocrine Neoplasia and Hormonal Disorders The University of Texas MD Anderson Cancer Center Houston, TX United States; 4 Department of Surgery University of California San Francisco San Francisco, CA United States; 5 Department of Head and Neck Surgery The University of Texas MD Anderson Cancer Center Houston, TX United States

**Keywords:** patient decision aids, decision support techniques, oncology, medullary thyroid cancer, targeted therapy, clinical trial, mobile phone

## Abstract

**Background:**

In cancers with a chronic phase, patients and family caregivers face difficult decisions such as whether to start a novel therapy, whether to enroll in a clinical trial, and when to stop treatment. These decisions are complex, require an understanding of uncertainty, and necessitate the consideration of patients’ informed preferences. For some cancers, such as medullary thyroid carcinoma, these decisions may also involve significant out-of-pocket costs and effects on family members. Providers have expressed a need for web-based interventions that can be delivered between consultations to provide education and prepare patients and families to discuss these decisions. To ensure that these tools are effective, usable, and understandable, studies are needed to identify patients’, families’, and providers’ decision-making needs and optimal design strategies for a web-based patient decision aid.

**Objective:**

Following the international guidelines for the development of a web-based patient decision aid, the objectives of this study are to engage potential users to guide development; review the existing literature and available tools; assess users’ decision-making experiences, needs, and design recommendations; and identify shared decision-making approaches to address each need.

**Methods:**

This study used the decisional needs assessment approach, which included creating a stakeholder advisory panel, mapping decision pathways, conducting an environmental scan of existing materials, and administering a decisional needs assessment questionnaire. Thematic analyses identified current decision-making pathways, unmet decision-making needs, and decision support strategies for meeting each need.

**Results:**

The stakeholders reported wide heterogeneity in decision timing and pathways. Relevant existing materials included 2 systematic reviews, 9 additional papers, and multiple educational websites, but none of these met the criteria for a patient decision aid. Patients and family members (n=54) emphasized the need for plain language (46/54, 85%), shared decision making (45/54, 83%), and help with family discussions (39/54, 72%). Additional needs included information about uncertainty, lived experience, and costs. Providers (n=10) reported needing interventions that address misinformation (9/10, 90%), foster realistic expectations (9/10, 90%), and address mistrust in clinical trials (5/10, 50%). Additional needs included provider tools that support shared decision making. Both groups recommended designing a web-based patient decision aid that can be tailored to (64/64, 100%) and delivered on a hospital website (53/64, 83%), focuses on quality of life (45/64, 70%), and provides step-by-step guidance (43/64, 67%). The study team identified best practices to meet each need, which are presented in the proposed decision support design guide.

**Conclusions:**

Patients, families, and providers report multifaceted decision support needs during the chronic phase of cancer. Web-based patient decision aids that provide tailored support over time and explicitly address uncertainty, quality of life, realistic expectations, and effects on families are needed.

## Introduction

### Background

As diagnoses and treatments continue to improve, chronic cancer is increasingly recognized as a unique phase in the cancer care continuum. During this time, many patients face difficult decisions such as whether to try novel therapeutics emerging on the market, whether to enroll in clinical trials, and when to stop treatment. New medicines offer hope but may provide only limited efficacy in select groups, have significant risks of side effects, or involve high out-of-pocket costs for the family. Many clinical trials cover the costs of treatment but involve accepting unknown potential benefits and risks. For some patients, even successful therapeutic effects do not last, and a decision needs to be made about whether to switch therapies or stop treatment. These decisions are classified as *preference-sensitive* because they involve 2 or more medically relevant options, uncertain benefits, notable risks, and variation in how patients and families value the potential process and outcomes [1].

A prime example is medullary thyroid carcinoma (MTC), a rare thyroid tumor. More than half of patients with MTC develop an advanced or chronic disease and live for years to decades with slowly progressing, often terminal cancer [2,3]. The US Food and Drug Administration approved 3 oral targeted therapies—vandetanib, cabozantinib, and selpercatinib—for the treatment of progressive MTC. Large phase 3 trials comparing vandetanib and cabozantinib with placebos showed improved progression-free survival; however, improvement in overall survival was only observed in small select groups [4,5]. These drugs are costly (US $200-US $600 per day) and can cause significant diarrhea, weight loss, hypertension, hypertensive crises, profound fatigue, or death [4,6]. Selpercatinib has been recently approved for a subset of patients and has been reported to be well tolerated with fewer side effects; however, overall survival benefits have not been shown [7]. Several clinical trials are ongoing; however, patients must be willing to accept randomization and unknown side effects. When discussing a new targeted therapy or clinical trial, it is also important to clarify the conditions under which patients would want to switch or end treatment. These decisions are often revisited iteratively over months or years, with much of patients’ deliberations occurring between clinical consultations. Hence, providers have expressed interest in web-based approaches to helping patients and families learn about and prepare to discuss these preference-sensitive decisions [8].

In preference-sensitive care, the best decision involves integrating medical evidence and informed patient preferences. Shared decision-making interventions such as decision coaching and patient decision aids are the gold standard for optimal preference-sensitive care [9-11]. Decision coaching involves semistructured discussion to ensure that patients are well informed, have realistic expectations, are clear about their decision-making values, and have appropriate resources and support to implement the mutually agreed upon choice [12]. Patient decision aids are tools that provide up-to-date, balanced evidence about the options and activities to promote preparation for decision making, values clarification, communication, and engagement [13]. Patient decision aids may be delivered before, during, or between consultations. Multiple Cochrane Collaboration systematic reviews report that patient decision aids help patients become well informed, form more realistic expectations, clarify which risks and benefits matter most to them, and prepare for discussing these decisions with their clinical teams [13-15]. Decision counseling and patient decision aids also address patients’ decisional conflict, a state of anxiety that blocks taking action [16]. Previous studies have reported that for each point increase on the 0-100 Decisional Conflict Scale, patients were 23 times more likely to delay their decision, 59 times more likely to change their mind, 5 times more likely to express decision regret, and 19% more likely to blame doctors for bad outcomes [17].

### Objectives

The International Patient Decision Aid Standards Collaboration provides guidelines for evidence-based systematic development of patient decision aids [18], including reviewing the literature for up-to-date clinical information, assessing the quality and relevance of any existing tools, and conducting a formal decisional needs assessment to identify high-priority decision-making needs. The guidelines chapter, *Delivering Decision Aids Using the Internet* [19], also recommends a user-centered design process to ensure that the tools are accessible, usable, and meaningful. The long-term goal of this research program is to develop patient- and provider-facing decision support tools for chronic cancer. As a first step, the objectives of this study are to (1) engage users in a stakeholder advisory panel to guide development; (2) review the existing literature and available tools; (3) assess the decision-making experiences, needs, and recommendations of patients with MTC, family members, and providers; and (4) identify shared decision-making approaches to address each need.

## Methods

### Conceptual Framework

**Figure 1:**
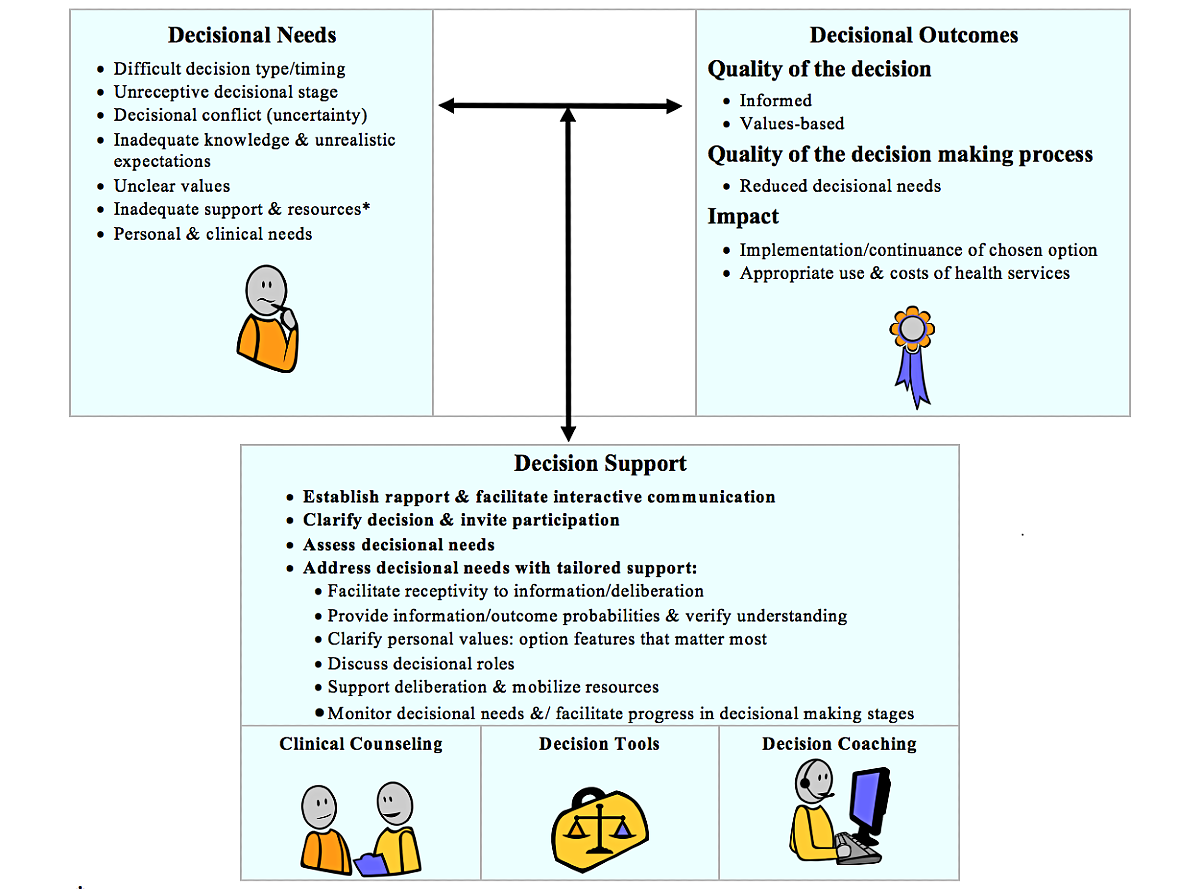
The Ottawa Decision Support Framework. *Inadequate support and resources to make/implement the decision include: information inadequacy/overload; inadequate perceptions of others' views/practices; social pressure; difficult decisional roles; inadequate experience; self-efficacy, motivation, skills; inadequate emotional support, advice, instrumental help; and inadequate financial assistance, health/social services. Copyright 2019, Ottawa Hospital Research Institute [16,20].

This initial study (part of a larger trial; NCT03892993) followed the multidisciplinary decisional needs assessment approach outlined by the patient decision aid development guidelines [18,19]. One of the key theoretical models underlying this approach is the Ottawa Decision Support Framework [16,20], which has been used to develop decision support interventions in more than 100 studies across 18 countries. This framework applies behavioral, cognitive, and economic decision theories [21-24] to preference-sensitive health care decisions. For example, it postulates several modifiable decision-making needs such as lack of awareness, knowledge, clarity, or support that can be addressed to ensure a high-quality decision-making process (Figure 1).

To support rigorous systematic development, this framework was operationalized in 1999 as the Decisional Needs Assessment in Populations [25]. This approach has been used across a wide variety of clinical contexts to assess patients’, families’, providers’, and community members’ decision-making experiences, processes, unmet needs, and recommendations for designing meaningful, understandable, and feasible tools [18,25-29]. It involves a series of steps using mixed methods with an emphasis on user-centered design and practical thematic analysis focused on unmet decision-making needs.

### Procedures

#### Overview

**Figure 2:**
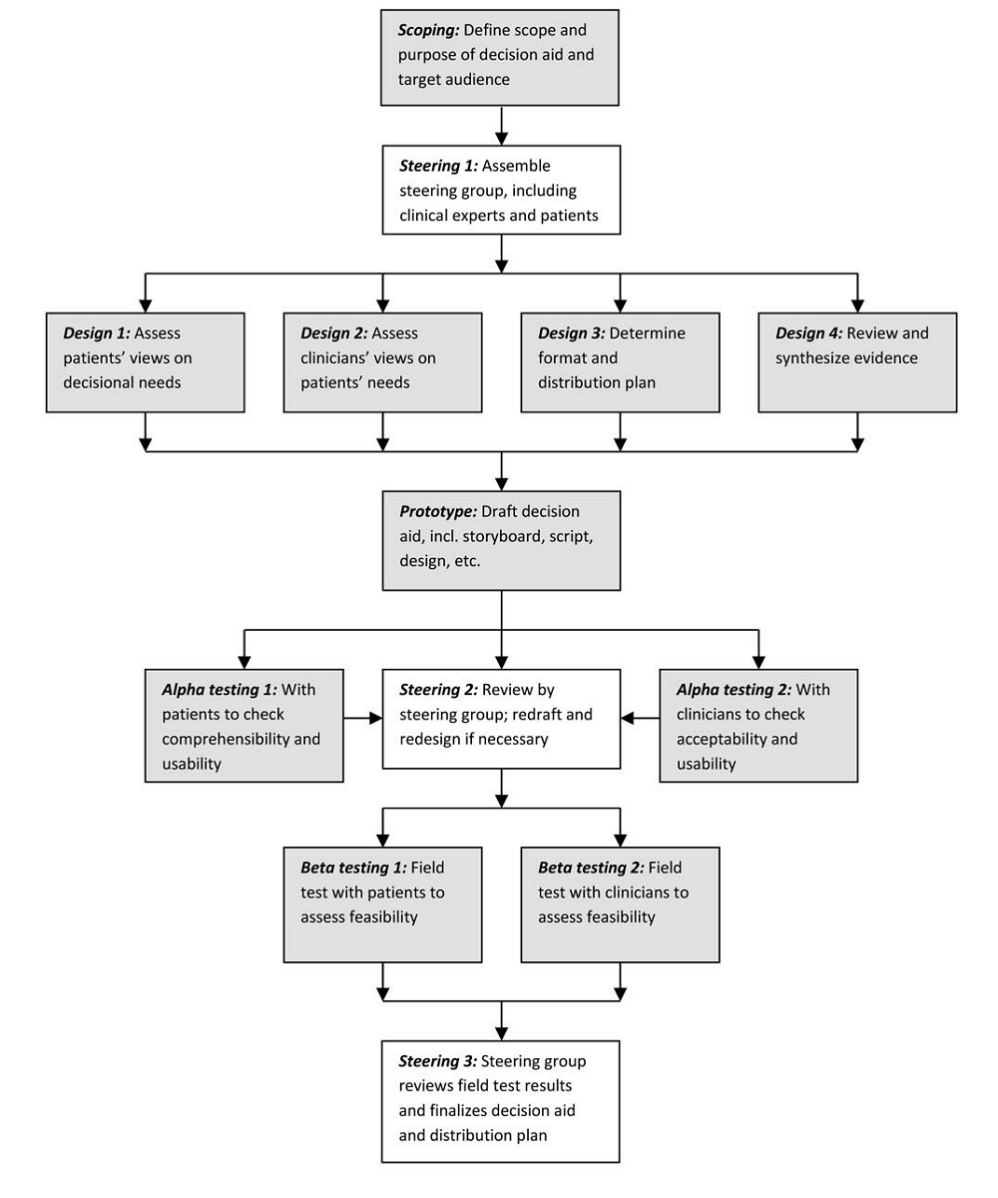
The International Patient Decision Aid Standards Collaboration model for the systematic development of a patient decision aid [18].

Following the guidelines for systematic development [18] (Figure 2) and delivery using the internet [19], this study proceeded in 4 steps: (1) engaging a stakeholder advisory panel, (2) reviewing existing literature and tools, (3) administering a decisional needs assessment questionnaire, and (4) developing a decision support design guide to inform the future design of a patient decision aid. The institutional review board of the University of Texas MD Anderson Cancer Center provided ethical review and approval.

#### Engaging Users in a Stakeholder Advisory Panel

To guide the overarching program of research, the study team purposefully invited a diverse group of potential users and key stakeholders, including patients, family members, surgeons, oncologists, and advanced practice providers. Advisory panel members were not participants but partners in the research process who provided guidance on the study methods, proposed questionnaire, potential sources, data interpretations, and the final design guide. The advisory panel meets at least twice per year to discuss the overarching program of research and was engaged in 2 study planning calls and multiple calls and emails as needed to review, edit, and approve study documents, results, interpretations, and this manuscript. Notably, they proposed that the project scope includes both targeted therapies and clinical trials as well as recommendations for a web-based patient decision aid to deliver timely information to patients living in the community.

#### Review of Existing Literature and Tools

A total of 2 reviews of MTCs had been completed in 2016 [30,31]. Therefore, a scoping review [32] was conducted to identify newer publications, and an environmental scan approach [33] was used to identify and assess the quality of existing materials. First, the research team members searched Web of Science, PubMed, and Google Scholar for *advanced medullary thyroid carcinoma*, *decision support*, *patient decision aid*, *vandetanib*, *cabozantinib*, *surveillance*, and *clinical trial participation*. Next, they used Web of Science to conduct a 1-generation forward and backward citation analysis of the references of the 2 systematic reviews and the papers that referenced the systematic reviews. Finally, the team conducted a gray literature search [34], which involved reviewing relevant websites, brochures, drug labels, and infographics using Google Scholar; web-based patient decision aid libraries; and relevant clinical, advocacy, and survivor support group websites. All reviews were conducted in English, included all time frames and countries, excluded advertisements, and retained only the most recent version of the edited documents or websites.

#### Decisional Needs Assessment

The decisional needs assessment questionnaire [25] includes 10 open-ended items assessing respondents’ previous experience, decision-making needs, and recommendations for the design of a decision support intervention such as a web-based patient decision aid. We tailored the questionnaire for patients, family members, and providers (eg, “Which of the following decisions [have you considered/have you discussed with your loved one/have you discussed with patients]?”). Patients and family members also responded to 14 items assessing their characteristics. To inform future implementation, providers responded to 2 questions about care pathways. The stakeholder advisory panel and institutional review board reviewed, revised, and approved the questionnaire.

The participants were English-speaking adult patients with MTC, informal caregivers or family members (on their own or with a patient or survivor), and clinicians who treat patients with MTC. The research coordinator recruited eligible patients and family members from the MTC Registry, which includes more than 1500 individuals from 20 states and 6 countries. The study team purposefully recruited patients across the disease spectrum, excluding individuals for whom participation could have caused distress (eg, recently diagnosed or bereaved). The provider participants included endocrinologists, medical oncologists, and surgeons purposefully recruited for their significant expertise and diversity of perspectives. All participants provided informed consent.

Members of the MTC Registry are active on the web and well known to each other and to MTC providers; therefore, additional attention was given to ensuring confidentiality while maximizing access. The research coordinator called each individual and offered an informational email that explained the purpose of, and process for, participating, including the opportunity to respond confidentially and securely on the web (or on paper or by phone, if requested). The email provided the link to a closed web-based questionnaire hosted on REDCap (Research Electronic Data Capture; Vanderbilt University) version 9.1.0 (May 31, 2019) [35]. A total of 2 reminder emails were sent to the nonresponders. All participants completed the questionnaire between May and September 2019. Patients and family members received a US $10 gift card after participating.

### Statistical Analysis

This exploratory study was not designed to test a hypothesis or generate a theory. Sample sizes were based on the international guidelines and were consistent with previous studies [18,19,28,36-39]. For the questionnaire, the research team used descriptive statistics to summarize quantitative responses and semantic, critical realist thematic analyses [40] to analyze qualitative responses. Beginning with a deductive approach, one author (ASH) hand-coded responses using core concepts from the Ottawa Decision Support Framework [16] (eg, uncertainty and need for information) and clinical literature (eg, side effects and costs). If a new concept arose in 2 or more responses, a new code was proposed, reviewed by a second author (DZS), and used to recode previous records. Conflicts and questions were discussed by 2 authors (EGG and MIH). The full research team met and reviewed the coding and findings twice and then presented all results and findings to the advisory panel to confirm meaningful interpretation and contextualization.

### Decision Support Design Guide

The purpose of a decision support design guide is to identify top-priority design needs (ie, clinical content, decision support activities, graphics, delivery etc) and to propose design solutions to address each need. Consistent with the conceptual framework, the research team organized the needs according to the modifiable factors that contribute to high decisional conflict (ie, feeling uninformed, unclear, unsupported, uncertain, and ineffective) [16] and added a delivery or accessibility category focusing on web-based delivery. Responses were retained if they were top rated or most endorsed across both groups. To identify best practices in decision support and patient decision aid design, the research team consulted the International Patient Decision Aid Standards chapters [12,19,41-46], the Cochrane Collaboration reviews [13,14], selected decision support experts, and the advisory panel. The team aligned the best practices parallel to each need to create a decision support design guide.

## Results

### Engaging Users in a Stakeholder Advisory Panel

The stakeholder advisory panel consisted of 4 patients and family members, 2 oncologic endocrinologists, 1 head and neck surgeon, and 1 advanced practice provider. Their experience and diverse perspectives complemented the expertise of the research team, which included a surgical oncologist, an oncologic endocrinologist, a decision scientist, and a trained research assistant. The advisory panel participated meaningfully in all aspects of the study, from the protocol design to writing this manuscript.

### Review of Existing Literature and Tools

In addition to the previous 2 systematic reviews [30,31], the scoping review and citation analysis identified 9 papers specific to decision making for MTC [3,47-53]. Two papers focused on improving diagnosis and staging, 6 papers reported studies of targeted therapeutics, and the remaining paper provided updated clinical practice guidelines. No studies on patient decision aids for starting or stopping targeted therapies were identified. A total of 3 studies of patient decision aids for clinical trial enrollment exist in other contexts [54-57], along with a conceptual framework for development [58]. The environmental scan and gray literature search identified 56 blogs, websites, and posts by clinical and advocacy groups. Review of these webpages confirmed that patients were asking about targeted therapy and clinical trial enrollment decisions; however, no patient decision aid for MTC was identified.

### Decisional Needs Assessment

#### Participant Characteristics

The research team invited 106 patients, family members, and providers. A total of 74 individuals responded, and 64 (87%) individuals completed the questionnaire, including 46 (72%) patients, 10 (16%) family members, and 10 (16%) providers. Table 1 summarizes the respondents’ characteristics. In addition, 46% (25/54) of patients and family members received surgery and care at both the study site and outside institutions. Notably, the respondents included individuals who had recently undergone a secondary surgery or focal treatment, individuals with indolent disease who may face these decisions in the future, and individuals experiencing an advanced stage of disease treated with at least one systemic therapy agent. Many providers (6/10, 60%) reported being attending physicians who saw 30-50 patients with MTC per year.

The following paragraphs summarize participants’ responses to the 3 sections of the questionnaire assessing (1) previous decision-making experiences, (2) decision support needs, and (3) recommendations for designing a decision support tool such as a web-based patient decision aid. To protect individuals’ privacy, patient and family member responses have been combined, identifying information has been redacted in the quotes, and results from fewer than 5 patients and family members are reported but not quantified.

**Table 1:** Participants’ characteristics (N=64).

Characteristics			Patients and family members^a^ (n=54)		Providers (n=10)
Number of years of treating or being with patients with medullary thyroid carcinoma, median (minimum, maximum)			4.5 (<1, 24)		13 (5, 25)
**Location of cancer care or clinical practice, n (%)^b^**					
	MD Anderson Cancer Center	44 (95)		1 (10)	
	Another institution	27 (56)		9 (90)	
**Experience with medullary thyroid carcinoma, n (%)^b^**					
	Patient with medullary thyroid cancer or survivor	46 (85)		—^c^	
	Caregiver or family member	10 (19)		—^c^	
	Medical oncologist	—^c^		5 (50)	
	Endocrinologist	—^c^		4 (40)	
	Surgeon	—^c^		1 (10)	
Age (years), median (minimum, maximum)			52 (21, 80)		46 (40, 60)
Female sex, n (%)			30 (56)		2 (20)
**Race, n (%)^b^**					
	White	46 (85)		7 (70)	
	Other	8 (15)		3 (30)	
Hispanic or Latino/Latina, n (%)			7 (13)		0 (0)
**Education, n (%)**					
	Some college, associate’s or technical degree	14 (26)		—^c^	
	Bachelor’s degree	25 (46)		—^c^	
	Graduate degree	11 (20)		—^c^	
**Religion, n (%)^b^**					
	Atheism or agnosticism	5 (9)		—^c^	
	Christianity	46 (85)		—^c^	
**Health insurance, n (%)^b^**					
	Private insurance	49 (91)		—^c^	
	Medicare or Medicaid	17 (32)		—^c^	
**Annual household income (US $), n (%)**					
	<50,000	12 (22)		—^c^	
	50,001-100,000	12 (22)		—^c^	
	>100,000	30 (56)		—^c^	

^a^To protect patients’ privacy, cells with <5 responses have not been reported; therefore, not all sections total 100%.

^b^Participants could select more than 1 response.

^c^Not assessed in this study group.

#### Patient and Family Member Perspectives

Of the 54, 20 (37%) of patients and family members reported making decisions about whether to start a new targeted therapy drug, 24 (44%) had deliberated about whether to enroll in a clinical trial, and 9 (16%) had chosen to stop a therapy. *Other* responses included whether to take or increase synthetic thyroid hormone, undergo radiation therapy, or undergo surgery with the possibility of losing their voice. A few patients focused on personal decisions such as when to disclose their diagnosis, discuss cascade genetic testing, or inform their family about progression. One person highlighted the decision to accept that they had terminal cancer. Others described logistical decisions such as whether to travel for second opinions, treatment, or surgery:

Choosing doctors & treatment options to best suit my specific needs is difficult because they’re few and far between. There are different types of this rare cancer (inherited and sporadic) and...different gene mutations...which ultimately contribute to compartmentalizing and/or reducing treatment options and adversely impacting [one’s] specific needs.

Patient

Where to get help? Local endocrinologists and surgeons had little to no experience with MTC and even their comments were unsettling. Is there a benefit to traveling for treatment?

Patient

Most of the patients (31/44, 72%) reported worry as a primary barrier, including significant concern regarding how each option would affect their family financially, emotionally, and in caregiving burden. Some patients (12/54, 22%) reported feeling rushed or pressured (by their doctor, family, or insurance company) or worried about disappointing their doctor (10/54, 19%). A few patients reported challenges with trust and communication:

I had to decide if I wanted to fight my initial endocrinologist about having genetic testing. He told me it was very expensive and since my children were adopted it wasn’t critical. When I asked about my siblings, he shrugged.

Patient

Both patients and family members (16/54, 30%) focused on the need for help dealing with uncertainty, clarifying risks, and weighing future effects on quality of life. Some participants (12/54, 22%) reported information barriers such as difficulty finding trustworthy information or frustration over finding conflicting information. A few participants noted that they needed time to process information and to connect with survivors to discuss the lived experience. Notably, most patients (45/54, 83%) and family preferred “discussing treatment options and my preferences with my providers, then making the decisions together.” Only a few wanted to make the decision themselves (6/54, 11%) or have the doctor make the decisions (3/54, 6%):

[We] have small children...[so we] had to weigh in the travel and cost for our family.

Caregiver or family member

[I] was not allowed time to gain knowledge of MTC. Also, I did not seek a second opinion which would have been valuable.

Patient

Table 2 summarizes patients’ and family members’ ratings of possible features for a decision support tool. Overall, most of the patients and family members recommended using plain language and providing a step-by-step guide, example questions, charts, and a printable summary. *Other* recommendations included providing a glossary, responses to frequently asked questions, question-prompt list, tips for talking with employers, guidelines on how to select a provider, and activities to elicit and clarify what is most important in these decisions (eg, travel costs, financial considerations, and timing):

Keep the language simple. The video we watched for [a previous clinical trial] was very informative. We followed it just fine, but my thoughts drifted to those that might not have a high education level, how well would they comprehend?

Caregiver or family member

Keep a library of historical real Questions & Answers made by people.

Patient

Mental health information—particularly aimed at grief and how to include children. Explain mortality rates in lay person terms.

Patient

**Table 2:** Patients’ and family members’ recommendations for a decision support tool (n=54).

Recommendations for a patient decision aid	Patients and family members, n (%)
Explain each treatment option in plain language	44 (82)
A step-by-step guide to walk you through considering the decision	36 (67)
Example questions to ask the doctor	32 (59)
Charts comparing options side by side	30 (56)
Printable summary of your information at the end	28 (52)
Stories from other patients or families about what each option was like	27 (50)
A glossary of the medical terms	27 (50)
Keep it simple	26 (48)
Stories from other patients or families about how they made these decisions	25 (46)
Graphics that illustrate the risks (eg, 8 of 10,000 people)	25 (46)
Activities to help you sort out what is most important to you personally	16 (30)
A place to write down your questions for the doctor	14 (26)
Other	6 (11)

Regarding the amount of information, a slight majority (32/54, 59%) reported wanting “the key facts and lots of details about all of the options,” but others preferred “the key facts, plus detail about the options I am interested in” (12/54, 22%) or “the key facts about each of the treatment options” (10/54, 19%). A few participants commented that they particularly wanted more information for these decisions because of the potential long-term effects on quality of life and on their families. Notably, a few participants requested information comparing clinical trials and information comparing novel therapeutics:

A simple pros and cons list for each clinical trial...or, a chart where you can see each drug side-by-side.

Patient

Regarding preferred mediums, most of the patients and family members recommended a worksheet that walks them through the decision(s) (39/54, 73%), an interactive website (35/54, 66%), a 1-page printable summary (34/54, 64%), or video (30/54, 57%). Most of the participants recommended multiple delivery routes, including the hospital website (45/54, 83%), personal email (37/54, 57%), smartphone app (25/54, 46%), paper copies at the doctor’s office (19/54, 35%), or mail (13/54, 24%). A few individuals recommended sending the worksheet through the patient portal, providing a link in the annual guidelines, or making it available on social media cancer support groups, peer-to-peer support groups, and patient advocacy sites.

#### Provider Perspectives

Regarding current experiences, all providers reported that their care pathway involves treatment by an endocrinologist until progression occurs, followed by referral to a medical oncologist. In total, 70% (7/10) of providers recommended introducing these decisions early on, including at the initial visit. In total, 20% (2/10) of providers recommended waiting until the patient developed distant disease:

[Targeted therapy should be discussed] as soon as distant metastases are identified. If they are small distant mets, then it is a brief mention that systemic therapy options are available in the future. As the mets get bigger or if they are progressing, then more detailed discussions ensue.

Endocrinologist

At the initial visit, I provide a comprehensive picture of their disease management, the palliative nature of therapy, the role of surveillance, what guides the decision to treat, and what the treatment options are (standard and experimental).

Medical oncologist

Regarding clinical trials, the providers reported engaging in discussions at least once a week (3/10, 30%), once a month (4/10, 40%), or a few times a year (2/10, 20%). Most of the providers (7/10, 70%) felt that it was their role to initiate these conversations with patients; others (3/10, 30%) felt that it could be introduced by the study team. In total, 40% (4/10) of providers reported that patients initiated these decisions approximately 50% of the time or more:

The best quality discussion happens over several visits...more as a continuing conversation, rather than a sudden surprise discussion that it is time to start systemic therapy tomorrow. This gives patients time to think about the information, involve family members, do their own research, and come prepared with better questions.

Endocrinologist

The providers reported moderate satisfaction with these discussions (10-point scale score: mean 7.5, SD 2.1; minimum=2, maximum=10). Their descriptions of the “best outcome of this conversation” were that patients understood the key information (6/10, 60%), including that trials are experimental, and that they interacted or asked questions (6/10, 60%) and stated that the decision aligned with goals of care (4/10, 40%):

Success is when a patient and his/her family feel like they have a good understanding of the risks/benefits of their decision and are comfortable with the path we choose together.

Medical oncologist

Success is they understand that clinical trials are experimental, we don’t know if they are better than standard of care, and the risks may not be completely known.

Endocrinologist

When discussing targeted therapies, the providers reported needing interventions that address preconceived notions (5/10, 50%), misinformation (4/10, 40%), and time constraints (4/10, 40%). A few described situations in which patients were informed that there is no cure, but they continued to believe that a cure may still be achievable. They also discussed 2 effects of misinformation and preconceived notions: (1) patients assuming that the side effects are negligible and not considering quality of life or (2) patients assuming the degree of side effects to be so harmful that they will not consider a certain therapy. *Other* barriers included lack of visual aids, poor retention, difficulties clarifying goals of care, fear and logistical challenges related to clinic flow, paucity of multidisciplinary approaches, and insurance coverage:

[Barriers include] preconceived notions from patients that approved therapies are too toxic and not efficacious; some patients want to dictate the way they should be treated.

Endocrinologist

The limitations imposed by busy clinics and limited time with patients is the biggest hindrance. These are complicated issues that require time with the patient to have a comprehensive discussion that is well-received with the patient.

Medical oncologist

When discussing decisions about clinical trials, most of the providers (9/10, 90%) also described situations in which patients stated unrealistic goals, overly optimistic assumptions, or beliefs that the trial would be curative. However, half of the providers (5/10, 50%) also discussed conversations in which patients distrusted pharmaceutical companies and did not want to be a “guinea pig.” *Other* barriers included lack of visual aids, concerns about randomization, worry about the unknown risks of side effects, and potential logistical challenges or costs:

[Patients believe] that we are just experimenting on them and that we have no idea whether it will work...there is a general mistrust of drug companies.

Endocrinologist

[Patients tell me] “I am a guinea pig,” or [they are] overly optimistic that the trial drug will help them [and have few side-effects].

Endocrinologist

The providers recommended a variety of approaches to designing a web-based patient decision aid. All providers (10/10, 100%) endorsed the need to include both decisions (targeted therapies and clinical trials), and most (7/10, 70%) endorsed having the ability to tailor or separate the decisions. Table 3 summarizes their ratings of the possible formats. *Other* responses included purposefully designing the patient decision aid for repeated discussions over time; face-to-face meetings with a midlevel provider to discuss side effects; and 1-page summaries of the disease, therapies, and trials:

Individualization is key.

Medical oncologist

In the absence of a curative systemic therapy, I think I would use a decision aid that incorporates standard of care and clinical trials.

Endocrinologist

**Table 3:** Perceptions regarding potential decision support tools (N=64).

How helpful would the following tools be?			Patients and family members (n=54)		Providers (n=10)
**Patient-facing tools, n (%)**					
	A 1-page comparison chart of the treatment options you can use in consultation	34 (64)		9 (90)	
	A brochure or video patients can be given before their appointment	30 (57)		8 (80)	
	A worksheet about preferences and values they complete that you can add to their electronic health record	39 (74)		7 (70)	
	A page on your institutional website	35 (66)		6 (60)	
	A face to face meeting with a nurse to talk about side effects	0 (0)		6 (60)	
	Other	8 (15)		7 (70)	
**Provider-facing tools, n (%)**					
	A brief training seminar on the current evidence	—^a^		7 (70)	
	A brief training seminar on decision coaching as a clinical skill	—^a^		7 (70)	
	A collaborative meeting between departments to discuss the upstream/downstream impacts of these decisions	—^a^		7 (70)	
	A study of the patients’ reported experience	—^a^		7 (70)	
	Other	—^a^		0 (0)	

^a^Not assessed in this study group.

In terms of the key facts that should be conveyed, most of the providers (7/10, 70%) focused on quality of life and repeating that targeted therapies may not prolong survival. Some also focused on balanced discussion, lack of known probabilities, acknowledging that experimental therapy may be better tolerated, emphasizing that goals of care change over time, and describing co-pays. A few providers cautioned that the decision aid should advise patients to make sure that their expectations, both expressed and implied, are realistic before enrolling in a clinical trial. One provider also recommended explicitly addressing the concept of altruistic volunteering:

A tutorial on the role of clinical trials in drug development and patient care to establish general background before a discussion would be helpful.

Medical oncologist

The providers recommended introducing clinical trials early on or during the first visit (4/10, 40%), at the same time as standard therapies (3/10, 30%), or at all stages (3/10, 30%). A few providers recommended waiting until standard therapy failed, before surgery, or when a novel therapy has compelling clinical data. Several providers brought up improving the overall decision-making process, including developing better patient education materials (3/10, 30%) and initiating multidisciplinary conversations earlier (3/10, 30%). *Other* recommendations included getting clarity about expectations, verbally encouraging patients to communicate side effects, and planning additional time for these conversations. They also recommended that the conversation be led by a clinician who is experienced in caring for patients with MTC:

[We need] better patient education materials aimed toward patients in their language.

Endocrinologist

[We need] to have MTC patients see knowledgeable medical oncologists earlier in the disease course, so it doesn’t feel like a defeat when they’re referred to us.

Medical oncologist

### Decision Support Design Guide

**Figure 3:**
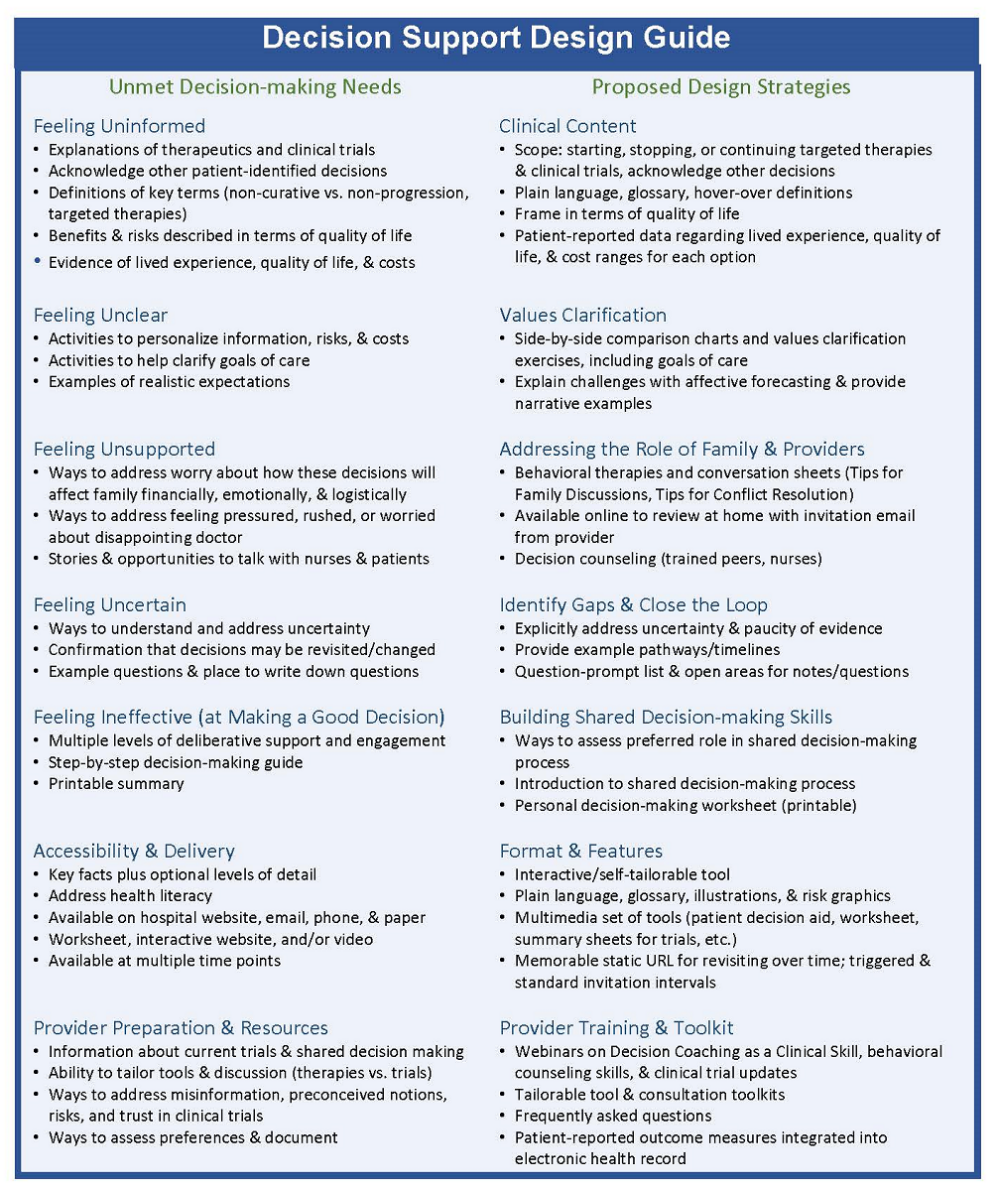
Decision support design guide. Left column: top-rated decision support needs from our assessment. Right column: proposed decision support strategies for addressing each need.

Figure 3 illustrates the decision support design guide, with the left column listing the top-reported decision support needs and the right column proposing decision support approaches to address each need. A review of the international standards identified several relevant best practices, including explicitly introducing shared decision making, inviting engagement, balanced presentation of pros and cons, addressing uncertainty, providing cost ranges, and delivering the patient decision aid on the hospital website with optional paper worksheets. To meet provider needs, strategies include seminars on decision coaching, communication, and behavioral therapy skills (to address anxiety, trust, fear, etc); a consultation tool kit of shared decision-making discussion prompts; responses to frequently asked questions; 1-page summaries of clinical trials; or illustrative material such as icon arrays and side-by-side comparison charts. The advisory panel reviewed and approved the design guide for use in future studies.

## Discussion

### Principal Findings

Overall, the results indicate that patients with chronic cancer may have significant unmet decision-making needs, and strong support exists for designing decision support tools regarding novel targeted therapies and clinical trial enrollment. Patients and family members report multiple information and decision support needs, such as needing understandable information, examples of the lived experience, help in personalizing the information, strategies to address worry, step-by-step guidance, and opportunities to revisit decision making over time. In addition, the providers emphasized the need to address misinformation, foster realistic expectations, and address mistrust toward clinical trials. The participants supported the development of a web-based tool that can be delivered across multiple platforms (hospital website and email) and that provides a printable personal summary. The providers also requested tools to support shared decision making in consultation. Although no existing patient decision aid could be identified, clinical content regarding targeted therapies is available, and examples of clinical trial tools exist in other clinical contexts. To support clinicians and designers who wish to develop such a tool, the proposed decision support design guide (Figure 3) illustrates the top-priority needs and best practices in decision support to address each need.

### Comparison With Previous Work

The results of this needs assessment highlighted several constructs, mechanisms, and behaviors that may affect patient decision aid design. Patients and family members reported heterogeneous information-seeking behaviors and deliberative styles. Some preferred “just the key facts” and may have been overwhelmed by too much detail, whereas others sought highly detailed information and side-by-side comparison charts. Some preferred implicit decision support (introducing the concepts of shared decision making but allowing them to manage their deliberative process internally), whereas others specifically requested explicit decision support (providing step-by-step guidance, interactive websites, or worksheets) [19]. These patterns are consistent with those in other studies [19,27] and may be linked to coping strategies such as monitoring (seeking a sense of control by seeking and attending to information) or blunting (seeking a sense of control by limiting information) [59,60]. Additional research is needed to explore dynamic designs that assess and address each patient’s information-seeking and deliberative styles.

Furthermore, these decisions highlight the challenges of shared decision making in the context of chronic or terminal stages of disease. The Ottawa Decision Support Framework focuses on addressing modifiable constructs to decisional conflict, such as feeling uninformed, unclear, and uncertain [16,17]. When discussing novel therapeutics and clinical trial enrollment, the options for providing evidence to foster certainty may be limited. However, the process of helping patients acclimate to the paucity of information, clarify what matters most (including quality of life), develop shared decision-making skills, and communicate with family and providers may provide important benefits that improve long-term decisional regret.

To our knowledge, this is the first study of MTC decision support needs and recommendations for a patient decision aid [13,61]. Previous studies have focused on the quality of patient-facing information. A review of 100 thyroid cancer websites [62,63] reported that most of the websites addressed diagnosis (92/100, 92%) and treatment (94/100, 94%); however, only some (50/100, 50%) were accurate, included source references (53/100, 53%), or were appropriate for a high school education level (2/100, 2%). A similar study in Germany [48] reported similar heterogeneity in the quality and accessibility of information. One study [50] tested web-based, personalized information and support with individuals with neuroendocrine tumors and found no difference in patients’ distress or satisfaction. A recent review of websites for the surgical management of low-risk thyroid cancer [64] reported that few (19/60, 32%) of the websites presented all treatment options, and none of the websites discussed the 2015 guidelines [3].

Since the 2017 review of internet-based patient decision aids [19], several studies have been published that may inform the design of web-based tools [65-72]. Overall, these studies continue to report positive ratings of acceptability, usability, and satisfaction as well as improved knowledge, decisional conflict, decision self-efficacy, preparation for decision making, and satisfaction. Baptista et al [73] reported that web-based patient decision aids improved knowledge and decisional conflict compared with usual care and were comparable with paper-based patient decision aids. Related reviews of computerized decision aids [74,75] report positive outcomes and satisfaction, strong correlations with the quality of development, and improved decision making for tools with features such as content control but poorer decision making when tools included tailoring or patient narratives. These topics continue to be emerging areas of research and can be explored in user design studies.

On the basis of the results of this study, the next steps will include continuing with the systematic development of a patient decision aid (Figure 2) for patients and family members with MTC. Once the review and synthesis of the clinical evidence are complete and we develop the initial prototype with the advisory panel, we will re-engage the participants who consented to be recontacted to iteratively review and revise the drafts. In parallel, we will develop and validate a Decision Quality Index, the gold standard measure of the degree to which patients’ decisions are informed and values congruent. With these tools, we can begin to simultaneously assess efficacy and effectiveness as well as explore some of the methodological research questions discussed herein. The ultimate long-term goals of this program of research continue to be to help patients with MTC and their families to make well-informed personal health care decisions, while simultaneously learning from patient-reported data about patients’ needs, values, cultural considerations, and informed preferences regarding starting and stopping novel therapeutics and clinical trials.

### Limitations

Several limitations of this study should be noted. The use of a web-based questionnaire typically limits follow-up; however, respondents are well known in this small and very active community, and most of the patients and family members consented to continued involvement in the iterative design and testing of tools and resources. Most patients received care at a comprehensive care center; however, we included patients across the spectrum of care (newly diagnosed to advanced disease), and responses were received from 19 states. This initial study focused on decisions about therapeutics and clinical trial enrollment; however, needs assessments are needed for the other difficult decisions identified by the respondents.

### Conclusions

Patients with chronic progressive cancers and their families face difficult decisions involving high uncertainty, complex topics, and concerns about potential effects on the family. High-quality patient decision aids are needed that provide information in plain language, explain how to make a decision under uncertainty, incorporate quality of life, address potential effects on family members, and can be revisited over time.

## Abbreviations

MTC: medullary thyroid carcinoma
REDCap: Research Electronic Data Capture
